# Uridine Metabolism and Its Role in Glucose, Lipid, and Amino Acid Homeostasis

**DOI:** 10.1155/2020/7091718

**Published:** 2020-04-14

**Authors:** Yumei Zhang, Songge Guo, Chunyan Xie, Jun Fang

**Affiliations:** College of Bioscience and Biotechnology, College of Resources and Environment, Hunan Agricultural University, Changsha, 410128 Hunan, China

## Abstract

Pyrimidine nucleoside uridine plays a critical role in maintaining cellular function and energy metabolism. In addition to its role in nucleoside synthesis, uridine and its derivatives contribute to reduction of cytotoxicity and suppression of drug-induced hepatic steatosis. Uridine is mostly present in blood and cerebrospinal fluid, where it contributes to the maintenance of basic cellular functions affected by UPase enzyme activity, feeding habits, and ATP depletion. Uridine metabolism depends on three stages: *de novo* synthesis, salvage synthesis pathway and catabolism, and homeostasis, which is tightly relating to glucose homeostasis and lipid and amino acid metabolism. This review is devoted to uridine metabolism and its role in glucose, lipid, and amino acid homeostasis.

## 1. Introduction

Uridine, a necessary pyrimidine nucleotide for RNA synthesis, can be synthesized *de novo* in mammals [1–3]. Uridine has been widely used in reducing cytotoxicity [4] and improving neurophysiological functions [5, 6]. In addition, short-term uridine coadministration with tamoxifen could suppress hepatic steatosis [2, 7–10]. In humans, uridine is present in the blood and cerebrospinal fluid, and the content of uridine in plasma is much higher than that in other purine and pyrimidine nucleosides [7, 11]. Due to their inability to synthesize uridine, most tissues utilize plasma uridine to maintain basic cellular functions [1, 12]. Thus, plasma uridine may be used for the synthesis of endogenous pyrimidine. The biosynthesis of uridine is regulated by the liver and adipose tissues, and the excretion of uridine is mainly achieved via the kidneys or by pyrimidine catabolism in tissues [13]. Many research studies have demonstrated that uridine homeostasis is affected by a variety of factors to regulate systemic metabolism. Factors recognized for regulating the concentration of uridine include uridine phosphorylase (UPase), feeding behavior, and ATP depletion.

In contrast, uridine can also regulate systemic homeostasis by regulating enzymes and their reaction products. Uridine is thought to regulate the mitochondrial respiratory chain via the dihydroorotate dehydrogenase (DHODH) enzyme, and since mitochondrial dysfunction is involved in many human diseases, uridine could be used as a therapeutic drug for mitochondrial diseases [10]. Furthermore, the accumulation of orotate, an intermediate product in the process of uridine *de novo* synthesis, induces intracellular lipid accumulation [14]. Accordingly, in this review, we focus on the factors that influence uridine concentration and the role of uridine in the metabolism of glucose, lipid, and amino acid.

## 2. Uridine Biosynthesis and Catabolism Pathway

It is acknowledged that pyrimidine nucleotide metabolism involves three pathways: *de novo* synthesis, salvage synthesis pathway, and catabolism. Within the cell, nucleotides can be synthesized from a simple metabolite by the *de novo* synthesis pathway or recycled by the salvage synthesis pathway [1]. When endogenous supply is insufficient to maintain normal body functions, uridine is mainly exogenously supplemented by diet to maintain normal growth and cell function [15]. During its catabolism, uridine is converted to *β*-alanine and followed by secretion to the brain and muscle tissues [1].

### 2.1. Uridine Biosynthesis

**Figure 1:**
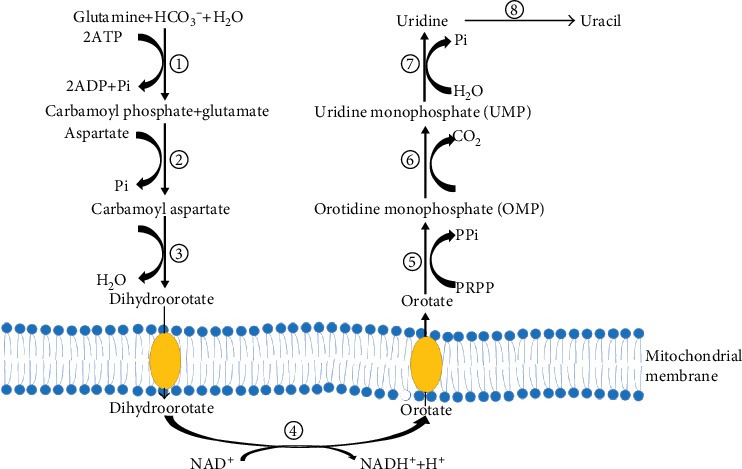
*De novo* synthesis of uridine: ① carbamoyl phosphate synthetase (CPS II); ② aspartate transcarbamoylase (ATCase); ③ dihydroorotase (DHO); ④ dihydroorotate dehydrogenase (DHODH); ⑤ orotate phosphoribosyltransferase; ⑥ orotidine 5′-phosphate decarboxylase; ⑦ nucleotidase; ⑧ uridine phosphorylase.

Uridine *de novo* synthesis originates from glutamine and is catalyzed by the CAD protein (which encodes the rate-limiting enzymes during uridine biosynthesis). CAD is a multidomain enzyme comprised of carbamoyl phosphate synthetase (CPS II, EC=6.3.5.5), aspartic transcarbamoylase (ATCase, EC=2.1.3.2), and dihydroorotase (DHO, EC=3.5.2.3), in which CPS II is the rate-limiting enzyme, and regulation of CPS II is mediated by uridine 5′-triphosphate (UTP) feedback inhibition and phosphoribosyl pyrophosphate (PRPP) activation [15]. In the *de novo* pathway, CAD catalyzed glutamine produces the intermediate metabolites carbamoyl phosphate, carbamoyl aspartate, and dihydroorotate (Figure 1).

The fourth step in pyrimidine biosynthesis is catalyzed by DHODH, to form an important intermediate, orotate. The accumulation of orotate may induce intracellular lipid accumulation [10]. DHODH is a mitochondrial, respiratory chain-coupled enzyme on the outer surface of the mitochondrial inner membrane, which links pyrimidine biosynthesis to mitochondrial energy metabolism [10, 15]. Then, orotate is converted to orotidine monophosphate (OMP) and uridine monophosphate (UMP) by orotate phosphoribosyltransferase and orotidine 5′-phosphate decarboxylase, respectively. Then, UMP is dephosphorylated to uridine by nucleotidase or formed from cytidine by cytidine deaminase (EC=3.5.4.5) [1, 10, 12].

A recent study has shown that resting/low proliferating cells rely mainly on the nucleotide salvage pathway to synthesize RNA [16]. Also, uridine can be obtained from UTP and cytosine 5′-triphosphate (CTP) via the pyrimidine salvage pathway. UTP is also involved in glycogen synthesis, protein glycosylation, and membrane phospholipid biosynthesis [17–19]. In the pyrimidine nucleotide salvage synthesis pathway, uridine-cytidine kinase 2 (UCK2) acts as a phosphorylase responsible for phosphorylating pyrimidine nucleotides (uridine and cytidine) to the corresponding monophosphates [15]. Nucleoside monophosphate kinase further phosphorylates UMP and CMP to produce uridine 5′-diphosphate (UDP) and cytosine 5′-diphosphate (CDP). UDP and CDP are further phosphorylated by the nucleoside diphosphate kinase to produce UTP and CTP. Subsequently, UTP and CTP are used only for gene duplication. During nucleic acid catabolism, some nucleoside monophosphates (NMPs) are released and reused in the salvage synthesis pathway by UCK2 [20]. In mammals, UCK2 is allosterically activated by ATP to synthesize the pyrimidine nucleotides required for cellular metabolism. Nucleotide production is controlled by the feedback inhibition of UCK2 by UTP and CTP [21]. Therefore, UCK2 is a potential chemotherapeutic drug target.

### 2.2. Uridine Catabolism

Uridine catabolism produces *β*-alanine and acetyl-CoA, resulting in an increase in protein acetylation [10, 22]. Uridine is degraded to uracil by uridine phosphorylase (UPase) encoded by the UPP gene (EC=2.4.2.3). UPase has two homologous forms in vertebrates: UPase1 and UPase2 [10, 11, 23]. UPase1, encoded by the UPP1 gene, regulates uridine homeostasis and is ubiquitously expressed. Inhibition of the enzymatic activity of UPase1 or UPase1 gene knockout results in elevated levels of uridine in plasma and tissues [11, 24]. UPase2 is a liver-specific protein encoded by the UPP2 gene and is indispensable for pyrimidine salvage reactions [2, 11]. Inhibition of the enzymatic activity of UPase2 increases the level of endogenous uridine in the liver, thereby protecting the liver against drug-induced lipid accumulation [2, 25]. Next, uracil is further decomposed into dihydrouracil and N-carbamoyl-*β*-alanine by dihydropyrimidine dehydrogenase (DPD) and hydropyrimidine hydratase (also known as dihydropyrimidinase), which is further converted to *β*-alanine by *β*-ureidopropionase (EC=3.5.1.6) [1, 10]. *β*-Alanine is excreted or enters other tissues, such as the brain and muscle.

## 3. The Regulation Mechanism of the Uridine Concentration

The plasma uridine concentration in mammals is between 3 and 8 *μ*M, which is higher than other pyrimidine nucleotides and bases [1, 26]. Most tissues cannot synthesize uridine; thus, use plasma as a uridine source [7, 11]. Plasma uridine concentration is tightly controlled by a variety of factors in both humans and rodents [1, 27]. However, there is no review of factors that regulate the concentration of uridine at present. Therefore, we next focus on factors that regulate the concentration of uridine and its mechanisms.

### 3.1. Feeding Behavior Regulates the Plasma Concentration of Uridine

A series of recent studies indicate that the dynamic regulation of plasma uridine is related to feeding behavior [11, 23]. As the main organ in uridine biosynthesis, the liver maintains uridine homeostasis by regulating glucose metabolism during different feeding states [12, 27]. In the fed period, uridine synthesis occurs mainly in the liver tissue. However, in the fasted period, uridine synthesis depends on adipocytes, as the liver focuses on glucose production [12]. Experiments to study how fasting and refeeding can cause changes in plasma uridine levels were performed in C57BL/6 mice, Sprague-Dawley rats, and healthy women. In the fasted state, adipose tissue elevated the plasma concentration of uridine via uridine biosynthesis, while plasma uridine levels decreased rapidly after refeeding [27]. The reduction is caused by a decrease in uridine synthesis in adipocytes and an increase in uridine clearance in bile [27–29].

Uridine can be catalyzed in vivo into uracil by uridine phosphorylase. It is hypothesized that uridine phosphorylase is a direct cause of the significant increase in uracil and dihydrouracil during the fasting state [26, 28]. Endogenous plasma uracil levels are largely dependent on uridine homeostasis rather than on food intake [28]. Thus, plasma uridine concentration is increased during fasting while decreased after refeeding.

### 3.2. Adipose Tissue Regulates Plasma Uridine Homeostasis

Recent studies have shown that adipose tissue plays an indispensable role in plasma uridine homeostasis. Plasma uridine can be elevated by pyrimidine degradation induced by ATP depletion. In FAT-ATTAC mice, a model in which adipocytes can be selectively eliminated by inducing apoptosis, placed under different feeding conditions, was selected to determine if adipose tissue is involved in the regulation of plasma uridine content during fasting [27, 30]. In addition, Agpat 2 (1-acylglycerol-3-phosphate-O-acyltransferase 2) selectively silenced (a mutant in which white fat and brown fat are absent) mice was studied to confirm that adipose tissue is essential for the rapid increase of plasma uridine in the fasting state [27, 31]. To explore the mechanism by which adipose tissue regulates plasma uridine homeostasis, researchers generated an adipose tissue-specific CAD knockout mouse as a model. Results show no increase of uridine levels in the CAD knockout mice after 24 hours of fasting, indicating that the increase of plasma uridine concentration induced by fasting is mediated by the biosynthesis of uridine via adipocytes [27]. These investigations demonstrate the possible molecular mechanism involving adipose tissue in the regulation of plasma uridine homeostasis.

### 3.3. ATP Depletion Increases Uridine Concentration

Numerous studies have shown that fructose, sucrose, ethanol, xylitol, and strenuous exercise may increase purine base (hypoxanthine, xanthine, and urate) concentration by increasing pyrimidine degradation after ATP depletion and enhancing adenine nucleotide degradation thereby increasing the concentration of uridine [32, 33]. Because UTP is produced by UDP phosphorylation using ATP as a phosphate donor, a decrease in ATP concentration leads to a decrease in phosphorylation of UDP to UTP, leading to an increase in UDP and UMP. An increase in ATP concentration thus accelerates the degradation of uracil nucleotides (UTP⟶UDP⟶UMP⟶uridine), increasing plasma uridine concentration [34, 35].

Previous studies have demonstrated that fructose may enhance the degradation of adenine and uracil nucleotides. Fructose is rapidly phosphorylated to fructose-1-phosphate (F-1-P) using ATP as a phosphate donor [1, 36]. While ATP is derived from the phosphorylation of ADP, the phosphorylation of ADP is accelerated by inorganic phosphate (Pi), resulting in a decrease of available Pi [36]. Due to the lack of Pi in the reaction, ATP is dependent on the inhibition of oxidative phosphorylation of ADP. Also, during the metabolism of fructose, AMP deaminase promotes the conversion of AMP to IMP, increasing IMP concentration, resulting in a decrease of adenine nucleotides and an increase in serum urate concentration [36]. Eventually, the uridine produced by the liver passes through the hepatic vein into the blood, resulting in an increase in blood uridine concentration [1, 37]. Therefore, ingesting food and beverages that contain large amounts of fructose may help increase the plasma uridine concentration.

On the other hand, exercise can also increase plasma uric acid concentration by promoting the degradation of adenine nucleotides and the production of lactic acid in muscles [32, 38]. The final degradation product of adenine nucleotide, hypoxanthine, is released from the muscle into the blood and then transported into the liver, where it is converted into uric acid under the action of xanthine dehydrogenase [36]. Furthermore, plasma urate concentration is increased during and after strenuous exercise. Exercise-induced adenine nucleotide degradation is characterized by an increase in the concentrations of plasma hypoxanthine, jaundice, and uridine. The uric acid produced by the liver is released into the blood, thereby increasing the concentration of plasma urate [36].

Ethanol can also promote the degradation of uracil nucleotide by increasing the consumption of ATP, thereby increasing the concentrations of plasma urate and uridine. Purines also serve to increase the concentration of plasma uric acid [1, 39]. During alcohol metabolism, ATP is rapidly consumed and decomposed to uric acid. According to previous studies, there are two mechanisms for the acceleration of ethanol-induced uracil nucleotide degradation: one is the increase in ATP consumption, and the other is the decrease in ATP production [34, 35]. Ethanol can also increase the excretion of hypoxanthine and jaundice in urine by accelerating the degradation of adenine nucleotides and weakening the activity of xanthine dehydrogenase [39]. Accordingly, it is strongly suggested that fructose, ethanol, and strenuous exercise can increase the concentration of uridine by increasing pyrimidine degradation after ATP depletion.

## 4. Relationships among Uridine and Glucose, Lipid, and Amino Acid Metabolism

Uridine is associated with glucose homeostasis, lipid metabolism, and amino acid metabolism by regulating enzymes and intermediates in uridine metabolism, such as UTP, DHODH, and UPase, which further are involved in systemic metabolism. The pyrimidine ring of uridine is susceptible to glycosylation [40]. Uridine catabolism can also lead to an increase in cellular acetyl-CoA pool, which in turn increases protein acetylation [10]. Our previous studies showed that oral administration of uridine stimulated intestinal development, promoted nucleotide transport, improved growth performance, and regulated the fatty acid composition and lipid metabolism in weaned piglets [41–44]. We have demonstrated that maternal dietary supplement UR contributed to reducing the occurrence of diarrhea by regulating cytokine secretion and intestinal mucosal barrier function of suckling pigs [45]. A recent study demonstrated that uridine was able to inhibit stemness of intestinal stem cells in 3D intestinal organoids and mice [46].

### 4.1. Uridine and Glucose Homeostasis

As the UTP precursor, uridine can activate glycogen synthesis [6]. It was found that the injection of uridine increased the levels of both uridine diphosphonate- (UDP-) glucose and uridine diphosphonate-N-acetylglucosamine (UDP-GlcNAc) in skeletal muscle. Injection of uridine also caused significant insulin resistance, suggesting that the decrease of insulin levels induced by uridine is mediated by muscle UDP-N-acetylhexosamine accumulation [1, 33]. In addition, a previous study indicated that plasma uridine is a marker of insulin resistance in patients with noninsulin-dependent diabetes mellitus (NIDDM) [1]. The latest report also showed that chronic or short-term uridine supplementation in mice could lead to impairment in glucose tolerance and decreased insulin signaling [2, 29]. A study where wild-type mice fed with a high-fat diet and ob/ob mice were given uridine and glucose orally showed a significant improvement in glucose tolerance in wild-type mice, while no variation was observed in ob/ob mice [27]. Furthermore, intraperitoneal injection of uridine was carried out to verify the effect of uridine on glucose metabolism, which resulted in an improvement of glucose tolerance in wild-type mice, while deterioration in ob/ob mice [12, 27]. These findings indicated that leptin might mediate the effects of uridine on glucose metabolism. Another study in aging mice with insulin resistance confirmed the role of uridine, showing that uridine administration also improved glucose tolerance in aging mice [27].

Furthermore, uridine could affect body temperature and feeding behavior through glucose metabolism. High doses of uridine may reduce body temperature in rodents, and uridine may be a driving force for thermoregulation during fasting and refeeding [27], which is another strong validation that uridine is associated with glucose homeostasis.

### 4.2. Uridine and Lipid Metabolism

Several studies have revealed the relationship between uridine and lipid metabolism [2, 7, 10]. There are two possible mechanisms for the relationship between pyrimidine metabolism and lipid metabolism: one is inhibition of DHODH, a mitochondrial membrane-bound and respiratory chain-coupled enzyme. Inhibition of DHODH leads to microvesicular steatosis. The other mechanism is overexpression of the UPase1 enzyme, leading to the depletion of endogenous uridine levels [10]. Inhibition of DHODH may cause microvesicular steatosis, which can be alleviated after uridine supplementation, whereas uridine has no effect on DHODH enzyme activity in vitro. Other studies have also shown that supplementation with uridine induces changes in the liver's NAD^+^/NADH and NADP^+/^NADPH ratios and in the liver's protein acetylation profile, thereby inhibiting fatty liver development [10]. UPase1 and UPase2 catalyze the reversible conversion of uridine to uracil [1, 2, 10, 11]. UPase1 plays a significant role in the regulation of uridine homeostasis. Recently, a transgenic (knock-in) UPase1-TG mouse with a fatty liver phenotype was used to evaluate the effect of UPase1 overexpression on hepatic uridine homeostasis [10]. UPase1-TG mice exhibited depleted uridine concentration in the plasma and liver, which could be reversed by dietary uridine supplementation [10, 47]. In contrast, UPase1^−/−^ mice, with genetic knockout of the UPase1 gene, exhibited a decreased plasma and liver uridine concentration, which showed a protective effect on the multiple-drug-induced fatty liver [7–9]. Furthermore, enhancing endogenous hepatic uridine concentration by inhibiting UPase2 could suppress liver lipid accumulation induced by drugs [2].

The treatment period of uridine is intimately related to fatty liver development. The difference between the short- and long-term effects of uridine on liver lipid accumulation can be regulated by the homeostatic control of circulating uridine levels [2]. Short-term uridine treatment can prevent drug-induced hepatic lipid accumulation, while chronic uridine feeding may induce liver lipid accumulation and reduce systemic glucose intolerance [2]. Chronic uridine feeding leads to inhibition of liver-specific fatty acid-binding protein FABP1 expression, while repression of the expression of FABP1 is associated with a fatty liver in mice and humans, and chronic uridine feeding may be a driver of fatty liver [48, 49]. Also, recent studies have found that dynamic oral administration of uridine affects the rhythmic fluctuations of cholesterol, bile acid, lipid, and nucleotide metabolism-related genes [50–53]. Our previous study also showed that uridine supplementation at night regulated rhythmic fluctuations in cholesterol and bile acid metabolism genes by enhancing duodenal nucleotide transport and synthesis [50], which indicated that appropriate time of uridine treatment is critical to lipid metabolism. Undoubtedly, further studies are required to explore the specific mechanism of the effect of uridine on lipid metabolism.

### 4.3. Uridine and Amino Acid Metabolism

Amino acids have been shown to reduce uridine concentration, possibly through nucleoside transporters present in various cell types [33]. In an amino acid infusion study, amino acids did not affect hypoxanthine, xanthine, and uric acid plasma concentrations, while the content of plasma uridine decreased by 25.1% and 58.5% at 30 minutes and one hour after injection of amino acids, respectively [54]. Amino acid infusion can increase uric acid excretion in urine without affecting plasma uric acid concentration and excretion of hypoxanthine and jaundice, indicating that amino acids increase renal uric acid excretion through renal transport but do not affect the degradation of sputum, where glucagon may play an important role in the process of amino acid-induced intrarenal uric acid clearance [55].

Amino acids have also been demonstrated to reduce uridine plasma concentrations directly [1]. A previous study has shown that oral branched-chain amino acids (isoleucine, leucine, and valine) can reduce plasma uridine concentration without affecting plasma concentrations of purine bases (uric acid, hypoxanthine, and xanthine), glucagon, and insulin or the excretion of uridine and purines [1, 56, 57]. Studies have shown that branched-chain amino acids play an important role in nucleoside metabolism [58]. Branched-chain amino acids may reduce the concentration of plasma uridine without altering the concentration of insulin and glucagon; thus, it is suggested that branched-chain amino acids are the direct cause of the decrease in plasma uridine concentration, rather than a consequence of insulin and glucagon levels [56, 57]. However, the specific mechanism by which amino acid induces a decrease in plasma uridine concentration is still unclear.

Furthermore, aspartate as a precursor can provide direct carbon for *de novo* synthesis of pyrimidine nucleotides; aspartate and glutamate also provide a nitrogen source for pyrimidine and pyrene synthesis [15, 16]. Moreover, some glucogenic amino acids, including cysteine, serine, and arginine, can produce pyruvate acid, after which pyruvate enters the glucose metabolism process and is converted to glucose-6-phosphate (6-P-G), which then enters the pentose phosphate pathway to produce ribose-5-phosphate, and catalytic synthesis 5-phosphate ribose-1-pyrophosphate by PRPP pyrophosphate to participate in nucleotide metabolism [1, 59]. PRPP is required for *de novo* synthesis of pyrimidine and purine nucleotides, so the acceleration of *de novo* synthesis of PRPP may also increase the *de novo* synthesis of pyrimidine nucleotides, thereby accelerating pyrimidine nucleotide biosynthesis, followed by an increase of uridine [1].

Taken together, these results suggest that amino acids can provide a carbon or nitrogen source for pyrimidine nucleotide synthesis and influence uridine concentration via different metabolic pathways.

## 5. Conclusion

Uridine has biological effects on a variety of physiological processes, such as RNA synthesis, glycogen synthesis, and lipid deposition. Accordingly, these factors work together in order to regulate the concentration of uridine to maintain a relatively stable state. On the contrary, uridine is intimately related to the homeostasis of the organism, regulating glucose homeostasis, lipid metabolism, amino acid metabolism, and other life processes. In addition, because uridine has nontoxic drug components, it is currently used for the treatment of hereditary disease, cancer, seizure, and central nervous system disorders. Therefore, further clinical researches are needed to study the relationship between physiological and pathological functions of uridine and its clinical implications.

## References

[1] Yamamoto T., Koyama H., Kurajoh M., Shoji T., Tsutsumi Z., Moriwaki Y. (2011). Biochemistry of uridine in plasma. *Clinica Chimica Acta*.

[2] Urasaki Y., Pizzorno G., Le T. T. (2016). Chronic uridine administration induces fatty liver and pre-diabetic conditions in mice. *PLoS One*.

[3] Connolly G. P., Duley J. A. (1999). Uridine and its nucleotides:biological actions, therapeutic potentials. *Trends in Pharmacological Sciences*.

[4] McEvilly M., Popelas C., Tremmel B. (2011). Use of uridine triacetate for the management of fluorouracil overdose. *American Journal of Health-System Pharmacy*.

[5] Gallai V., Mazzotta G., Montesi S., Sarchielli P., Gatto F. (1992). Effects of uridine in the treatment of diabetic neuropathy: an electrophysiological study. *Acta Neurologica Scandinavica*.

[6] Mironova G. D., Khrenov M. O., Talanov E. Y. (2018). The role of mitochondrial KATP channel in anti-inflammatory effects of uridine in endotoxemic mice. *Archives of Biochemistry and Biophysics*.

[7] Le T. T., Urasaki Y., Pizzorno G. (2014). Uridine prevents fenofibrate-induced fatty liver. *PLoS One*.

[8] Le T. T., Urasaki Y., Pizzorno G. (2014). Uridine prevents tamoxifen-induced liver lipid droplet accumulation. *Bmc Pharmacology & Toxicology*.

[9] Lebrecht D., Vargas-Infante Y. A., Setzer B., Kirschner J., Walker U. A. (2007). Uridine supplementation antagonizes zalcitabine-induced microvesicular steatohepatitis in mice. *Hepatology*.

[10] Le T. T., Ziemba A., Urasaki Y., Hayes E., Brotman S., Pizzorno G. (2013). Disruption of uridine homeostasis links liver pyrimidine metabolism to lipid accumulation. *Journal of Lipid Research*.

[11] Cao D., Leffert J. J., McCabe J., Kim B., Pizzorno G. (2005). Abnormalities in uridine homeostatic regulation and pyrimidine nucleotide metabolism as a consequence of the deletion of the uridine phosphorylase gene. *The Journal of Biological Chemistry*.

[12] Greenhill C. (2017). Metabolism: liver and adipose tissue control uridine biosynthesis. *Nature Reviews Endocrinology*.

[13] Ison G., Beaver J. A., McGuinn WD Jr (2016). FDA approval: uridine triacetate for the treatment of patients following fluorouracil or capecitabine overdose or exhibiting early-onset severe toxicities following administration of these rugs. *Clinical Cancer Research An Official Journal of the American Association for Cancer Research*.

[14] Löffler M., Carrey E. A., Zameitat E. (2015). Orotic acid, more than just an intermediate of pyrimidine de novo synthesis. *Journal of Genetics and Genomics*.

[15] Vincenzetti S., Polzonetti V., Micozzi D., Pucciarelli S. (2016). Enzymology of pyrimidine metabolism and neurodegeneration. *Current Medicinal Chemistry*.

[16] Schoors S., Bruning U., Missiaen R. (2015). Fatty acid carbon is essential for dNTP synthesis in endothelial cells. *Nature*.

[17] Roach P. J., Depaoli-Roach A. A., Hurley T. D., Tagliabracci V. S. (2012). Glycogen and its metabolism: some new developments and old themes. *Biochemical Journal*.

[18] Vance J. E., Vance D. E. (2004). Phospholipid biosynthesis in mammalian cells. *Biochemistry and Cell Biology*.

[19] Pooler A. M., Guez D. H., Benedictus R., Wurtman R. J. (2005). Uridine enhances neurite outgrowth in nerve growth factor-differentiated pheochromocytoma cells. *Neuroscience*.

[20] Malami I., Abdul A. B. (2019). Involvement of the uridine cytidine kinase 2 enzyme in cancer cell death: a molecular crosstalk between the enzyme and cellular apoptosis induction. *Biomedicine & Pharmacotherapy*.

[21] Huang M., Graves L. M. (2003). De novo synthesis of pyrimidine nucleotides; emerging interfaces with signal transduction pathways. *Cellular and Molecular Life Sciences*.

[22] Urasaki Y., Pizzorno G., Le T. T. (2014). Uridine affects liver protein glycosylation, insulin signaling, and heme biosynthesis. *PLoS One*.

[23] Deliang C., Giuseppe P. (2004). Uridine phosophorylase: an important enzyme in pyrimidine metabolism and fluoropyrimidine activation. *Drugs Today*.

[24] Cao D., Ziemba A., McCabe J. (2011). Differential expression of uridine phosphorylase in tumors contributes to an improved fluoropyrimidine therapeutic activity. *Molecular Cancer Therapeutics*.

[25] Zhang Y., Repa J. J., Inoue Y., Hayhurst G. P., Gonzalez F. J., Mangelsdorf D. J. (2004). Identification of a liver-specific uridine phosphorylase that is regulated by multiple lipid-sensing nuclear receptors. *Molecular Endocrinology*.

[26] Pizzorno G., Cao D., Leffert J. J., Russell R. L., Zhang D., Handschumacher R. E. (2002). Homeostatic control of uridine and the role of uridine phosphorylase: a biological and clinical update. *Biochimica et Biophysica Acta (BBA)-Molecular Basis of Disease*.

[27] Deng Y., Wang Z. V., Gordillo R. (2017). An adipo-biliary-uridine axis that regulates energy homeostasis. *Science*.

[28] Henricks L. M., Jacobs B. A. W., Meulendijks D. (2018). Food-effect study on uracil and dihydrouracil plasma levels as marker for dihydropyrimidine dehydrogenase activity in human volunteers. *British Journal of Clinical Pharmacology*.

[29] Yang Q., Vijayakumar A., Kahn B. B. (2018). Metabolites as regulators of insulin sensitivity and metabolism. *Nature Reviews Molecular Cell Biology*.

[30] Pajvani U. B., Trujillo M. E., Combs T. P. (2005). Fat apoptosis through targeted activation of caspase 8: a new mouse model of inducible and reversible lipoatrophy. *Nature Medicine*.

[31] Cortés V. A., Curtis D. E., Sukumaran S. (2009). Molecular mechanisms of hepatic steatosis and insulin resistance in the AGPAT2-deficient mouse model of congenital generalized lipodystrophy. *Cell Metabolism*.

[32] Ka T., Yamamoto T., Moriwaki Y. (2003). Effect of exercise and beer on the plasma concentration and urinary excretion of purine bases. *Journal of Rheumatology*.

[33] Yamamoto T., Inokuchi T., Ka T. (2010). Relationship between plasma uridine and insulin resistance in patients with non-insulin-dependent diabetes mellitus. *Nucleosides, Nucleotides & Nucleic Acids*.

[34] Yamamoto T., Moriwaki Y., Cheng J. (2002). Effect of inosine on the plasma concentration of uridine and purine bases. *Metabolism Clinical and Experimental*.

[35] Yamamoto T., Moriwaki Y., Ka T. (2004). Effect of purine-free low-malt liquor (happo-shu) on the plasma concentrations and urinary excretion of purine bases and uridine--comparison between purine-free and regular happo-shu. *Hormone and Metabolic Research*.

[36] Ohno M., Ka T., Inokuchi T. (2008). Effects of exercise and grape juice ingestion in combination on plasma concentrations of purine bases and uridine. *Clinica Chimica Acta*.

[37] Kobayashi T., Inokuchi T., Yamamoto A. (2007). Effects of sucrose on plasma concentrations and urinary excretion of purine bases. *Metabolism*.

[38] Kaya M., Moriwaki Y., Ka T. (2006). Plasma concentrations and urinary excretion of purine bases (uric acid, hypoxanthine, and xanthine) and oxypurinol after rigorous exercise. *Metabolism*.

[39] Yamamoto T., Moriwaki Y., Takahashi S. (2005). Effect of ethanol on metabolism of purine bases (hypoxanthine, xanthine, and uric acid). *Clinica Chimica Acta*.

[40] Zhang Y., Knapp S. (2016). Glycosylation of nucleosides. *The Journal of Organic Chemistry*.

[41] Xie C., Wang Q., Li G., Fan Z., Wang H., Wu X. (2019). Dietary supplement with nucleotides in the form of uridine monophosphate or uridine stimulate intestinal development and promote nucleotide transport in weaned piglets. *Journal of the Science of Food and Agriculture*.

[42] Li G., Xie C., Wang Q. (2019). Uridine/UMP metabolism and their function on the gut in segregated early weaned piglets. *Food & Function*.

[43] Li B., Zhou H., Wu X., Chen Z., Yao J., Yin Y. (2016). Effects of dietary supplementation with uridine monophosphate on performance and intestinal morphology of weanling piglets1. *Journal of Animal Science*.

[44] Zhang Y., Guo S., Xie C. (2019). Short-term oral UMP/UR administration regulates lipid metabolism in early-weaned piglets. *Animals*.

[45] Wu X., Lu-min G., Yi-lin L. (2020). Maternal dietary uridine supplementation reduces diarrhea incidence in piglets by regulating the intestinal mucosal barrier and cytokine profiles. *Journal of the Science of Food and Agriculture*.

[46] Liu Y. L., Guo S. G., Xie C. Y., Niu K., De Jonge H., Wu X. (2020). Uridine inhibits the stemness of intestinal stem cells in 3D intestinal organoids and mice. *RSC Advances*.

[47] Le T. T., Ziemba A., Urasaki Y., Brotman S., Pizzorno G. (2012). Label-free evaluation of hepatic microvesicular steatosis with multimodal coherent anti-stokes Raman scattering microscopy. *PLoS One*.

[48] Martin G. G., Atshaves B. P., Landrock K. K., Landrock D., Schroeder F., Kier A. B. (2015). Loss of L-FABP, SCP-2/SCP-x, or both induces hepatic lipid accumulation in female mice. *Archives of Biochemistry and Biophysics*.

[49] Guzmán C., Benet M., Pisonero-Vaquero S. (2013). The human liver fatty acid binding protein (FABP1) gene is activated by FOXA1 and PPAR*α*; and repressed by C/EBP*α*: implications in FABP1 down-regulation in nonalcoholic fatty liver disease. *Biochimica et Biophysica Acta*.

[50] Zhang K., Liu Y. L., Zhang Y. (2019). Dynamic oral administration of uridine affects the diurnal rhythm of bile acid and cholesterol metabolism-related genes in mice. *Biological Rhythm Research*.

[51] Jacobs B. A., Deenen M. J., Pluim D. (2016). Pronounced between-subject and circadian variability in thymidylate synthase and dihydropyrimidine dehydrogenase enzyme activity in human volunteers. *British Journal of Clinical Pharmacology*.

[52] Liu Y., Zhang Y., Yin J., Ruan Z., Wu X., Yin Y. (2019). Uridine dynamic administration affects circadian variations in lipid metabolisms in the liver of high-fat diet-fed mice. *Chronobiology International*.

[53] Liu Y., Zhang Y., Yin J., Ruan Z., Wu X., Yin Y. (2019). Uridine dynamic administration affects circadian variations in lipid metabolisms in the liver of high-fat-diet-fed mice. *Chronobiology International*.

[54] Yamamoto T., Moriwaki Y., Takahashi S. (1999). Effect of amino acids on the plasma concentration and urinary excretion of uric acid and uridine. *Metabolism*.

[55] So A., Thorens B. (2010). Uric acid transport and disease. *The Journal of Clinical Investigation*.

[56] Yamamoto T., Moriwaki Y., Takahashi S. (2000). Effect of branched-chain amino acids on the plasma concentration of uridine does not occur via the action of glucagon or insulin. *Metabolism*.

[57] Zhang H., Fu P., Ke B. (2014). Metabolomic analysis of biochemical changes in the plasma and urine of collagen-induced arthritis in rats after treatment with Huang-Lian-Jie-Du-Tang. *Journal of Ethnopharmacology*.

[58] Schousboe A., Leke R., Bak L. (2008). Branched chain amino acids play an important role in ammonia homeostasis which, in turn, affects GABA biosynthesis. *Liver International*.

[59] Walker A. K. (2017). 1-Carbon cycle metabolites methylate their way to fatty liver. *Trends in Endocrinology and Metabolism*.

